# Novel t(5;19) Translocation in a Patient with PDGFRB Associated Chronic Leukemia: Implications for Treatment Strategy

**DOI:** 10.1155/2013/709164

**Published:** 2013-07-10

**Authors:** Joel D. Provenzano, J. Phillip Kuebler

**Affiliations:** ^1^Riverside Methodist Hospital, Columbus, OH, USA; ^2^Pulmonary Critical Care Medicine, 3901 Rainbow Boulevard, Kansas City, KS 66160, USA

## Abstract

Myeloproliferative disorders are variable disorders, based on the genetic abnormality present and the cell line progenitors that are affected. In this case, we discuss a novel gene translocation in the subset of PDGFRB mutations, first seen with prominent hyperbasophilia. This case demonstrates the possibility for lower therapeutic doses of imatinib mesylate than previously reported, in order to control leukocyte counts and reverse the genetic mutation.

## 1. Case Presentation

### 1.1. History

 J. H. is a 54-year-old male who initially presented to the emergency department with complaints of shortness of breath and peripheral edema, which had been occurring progressively over the preceding several days. His review of symptoms was also positive for an unintentional 30-pound weight loss and nightly sweats for at least three months. He had not seen a physician for many years, and his past medical history was positive only for chronic obstructive pulmonary disorder and a possible transient ischemic attack several years before this presentation. His family history was positive only for diabetes and hypertension. He admitted to a fairly heavy history of alcohol consumption and tobacco abuse.

### 1.2. Hospital Course

His initial workup targeted his heart failure symptoms, with a chest X-ray in the emergency department revealing pulmonary edema and an echocardiogram confirming a left ventricular ejection fraction of 35%. A computed-tomography scan of the abdomen revealed evidence of cirrhosis, with esophageal varices and splenomegaly. Leukocytosis was noted, with a leukocyte count of 52.57 K/mcL. He was also found to be anemic, with a hemoglobin of 7.6 g/dL, and thrombocytopenic, with a platelet count of 61,000.

 The patient responded well to diuresis and was stabilized from a cardiovascular and respiratory standpoint. Working diagnosis included both ischemic cardiomyopathy and alcoholic cirrhosis. His leukocytosis was initially thought to be reactive; however, his complaints of weight loss and night sweats prompted further investigation. Manual differential of his complete blood count revealed a left shift, with neutrophils 65%, lymphocytes 2%, monocytes 10%, eosinophils 7%, basophils 1%, metamyelocytes 12%, and myelocytes 3%. Iron studies, folate, and B12 levels were normal. Consultation to Hematology/Oncology was made. FISH for bcr-abl, along with t(9;22) and JAK-2, was sent to rule out CML and PRV. Peripheral blood for cytogenetics was also sent, with planned outpatient follow-up in the Hematology/Oncology clinic.

### 1.3. Follow-Up

 The patient was seen in follow-up two weeks later. A repeat WBC was 40.63 K/mcL, and the hemoglobin had decreased to 8.1 g/dL, despite transfusion as an inpatient and oral iron supplementation. His platelet count remained low, at 67,000. FISH for bcr-abl, t(9;22), and JAK-2 was negative. A bone marrow biopsy was performed, to check for myeloproliferative disorders. Peripheral blood smear revealed continued left-shifted neutrophilia with eosinophilia and basophilia. Hypersegmented neutrophils were also present. Bone marrow aspirate differential was the following: blasts 3%, promyelocytes 7%, myelocytes/metamyelocytes 36%, neutrophils 33%, eosinophils 7%, monocytes 3%, lymphocytes 2%, proerythroblasts 1%, basophilic erythroblasts 1%, and polychromatophilic erythroblasts 7%. The sample was noted to be hypercellular, with a myeloid to erythroid ratio of 10 to 1. Cytogenetic testing revealed the patient to have an abnormal karyotype in 15 of 20 mitotic cells evaluated. This karyotype was 46,XY,t(5;19)(q31;p13)[15]/46,XY[5]. Further cytogenetic analysis revealed the sample to be positive for a PDGFRB mutation as well.

### 1.4. Treatment Plan

 Previous evidence suggested some response in PDGFRB+ myeloproliferative disorders to imatinib mesylate. The patient was started on imatinib at a dose of 100 mg daily, and at the time of follow-up from the bone marrow biopsy, ten days later, his leukocyte count had dropped to 22.78 K/mcL, but with continued abnormal differential. Prior literature was unclear regarding optimal dosing for imatinib, ranging from 400 mg twice daily to 100 mg every other week. It was theorized that this patient could possibly be controlled with the lower dose, as his leukocyte count had begun to drop spontaneously. Within two weeks of initiation of his imatinib 100 mg daily, his leukocyte count had dropped to 13.57 K/mcL. He continued to have elevated monocytes, eosinophils, and basophils; however, his lymphocyte count showed signs of recovery, to 13.3%, and he had no evidence of myelocytes or metamyelocytes in the peripheral blood.

### 1.5. Response to Treatment

 On follow-up one month later, the patient had gained 10 pounds, and his energy and attitude were much improved. At the request of his hematologist/oncologist, he had stopped taking his imatinib several days prior, as his leukocyte count had returned to normal. Follow-up blood counts one month later showed his leukocyte count to be at the lower limit of normal, 5.2 K/mcL. His lymphocyte count had increased to 19%, and even though his monocyte count remained elevated at 14%, his eosinophil count and basophil count were at normal levels. No abnormal leukocytes were evident. Of note, his hemoglobin had improved to 10.9 g/dL, without transfusion, and his platelet count had improved to 102,000.

 Within one month of stopping his imatinib, the patient was found to again to have leukocytosis on a routine blood test. His leukocyte count was back up to 13.8 K/mcL, with evidence of 3% bands, 2% myelocytes, 4% metamyelocytes, and 1% promyelocytes in the peripheral blood. His lymphocyte count was down to 11%, his monocyte count remained elevated at 15%, and his eosinophil count had risen again to 6%. His hemoglobin had continued to improve, at 12.7 g/dL, and his platelet count remained essentially stable, at 95,000. It was decided to restart the patient on imatinib, at 100 mg daily. One month later, his leukocyte count had dropped again to 5.1 K/mcL. His differential was again near normal, with 19.9% lymphocytes, 9.7% monocytes, 1.4% eosinophils, and no early granulocytic precursors. Instead of discontinuing the imatinib, the dose was adjusted to 100 mg every other day, in an attempt to maintain a normal differential without producing leukopenia. One month later, a CBC showed a total leukocyte count of 6.29 K/mcL, with lymphocyte count now within normal range, at 26.9%, and normal eosinophil and monocyte counts. The hemoglobin was 13.0 g/dL, and the platelet count in this cirrhotic patient had stabilized around 100,000.

 Eighteen months after his initial presentation, the patient continued to have a normal leukocyte counts with no medication side effects. He is currently maintained on imatinib mesylate 100 mg three times per week.

## 2. Discussion

### 2.1. Overview

 Myeloproliferative disease is a bone marrow disorder, characterized by uncontrolled production by one or more of the hematopoetic progenitor cells. The myeloproliferative neoplasms (MPNs) include polycythemia vera, essential thrombocytosis, myelofibrosis, and chronic myelocytic leukemia. Classification of these diseases is based on the cell lines affected, which is determined by the genetic mutation or rearrangement present. The presence of the JAK-2 mutation suggests polycythemia vera, essential thrombocytosis, or myelofibrosis, while a positive BCR-ABL mutation is diagnostic for chronic myeloid leukemia. More recently, the identification of myeloid neoplasms with hypereosinophilia has been reported [1–3]. The most common genetic mutations observed in these disorders involve the platelet-derived growth factor receptor (PDGFR) and are further divided into PDGFRA and PDGFRB mutations.

The more common of the two PDGFR mutations involve PDGFRA on chromosome 4. PDGFRB mutations, seen in this patient, are more rare, involving the q31-31 loci on chromosome 5. Translocations associated with this gene can involve a number of different chromosomes, most commonly a t(5;12)(q31;p13) mutation. However, gene translocations involving 5q31-33 have also been identified with chromosomes 1, 2, 3, 4, 7, 10, 11, 12, 14, 15, 16, and 17 [2]. A unique translocation involving chromosome 19 was identified in our patient. An extensive search of the literature and genetic databases has failed to find another reported case of a PDGFR mutation associated with a translocation involving chromosome 19, as seen in this case.

 The translocated gene sequence from other chromosomes likely has little effect on the development of the disease. Previous case reports involving PDGFRB mutations have described features similar to chronic myelomonocytic leukemia, chronic eosinophilic leukemia, or mastocytosis [1, 2, 4]. Other reports have noted the presence of overlapping features of many different myeloproliferative neoplasms [5]. We report the first case involving a t(5;19) translocation, which appears to also feature prominent hyperbasophilia.

### 2.2. Role of PDGFRB

 The genetic abnormality in chronic leukemias with hypereosinophilia appears to be based entirely on the PDGFRB mutation. The PDGFRB locus has been reported to code for cleavage of PDGF ligands, and is expressed on both erythroid and myeloid precursors in the bone marrow. The expression on monocytic cells may indicate a role in the differentiation into more mature cells. As a result of PDGFRB mutations, the proteins are missing the binding domain with which to cleave the other PDGF ligands. The PDGFRB protein then becomes active itself, triggering other cell lines to proliferate. The most common association with this active PDGFRB protein involves the ETV6 gene, though over forty fusions have been discovered [2].

 The genes that code for eosinophilic differentiation are located in the same chromosomal region as the PDGFRB translocation, 5q31-q35. These genes include interleukin-3, interleukin-4, interleukin-5, interleukin-13, and granulocyte-macrophage colony-stimulating factor [6]. Some data suggests the specific involvement of interleukin-5 [1]. In some cases, morphologically normal eosinophils are found without translocations, which could mean that peripheral eosinophilia is instead a reactive process in these particular chronic leukemias [1]. There is also probably a role for PDGFRB in chemotaxis and signaling in the inflammatory cascade [1].

### 2.3. Clinical Findings

 MPNs associated with PDGFRB mutations have a broad range of clinical presentations, especially in those not involving t(5;12) [1]. However, previous reviews of the literature have identified some commonalities among patients with PDGFRB mutations. The most obvious are peripheral eosinophilia, splenomegaly, and a male predominance in the patient population [1, 7]. Onset is usually in the 6th-7th decade of life, though cases in children have been reported [6]. The pathologic diagnoses of PDGFRB associated mutations have included chronic myelomonocytic leukemia (CMML), chronic eosinophilic leukemia (CEL), atypical CML, myelodysplastic syndrome (MDS), and acute myeloid leukemia (AML) [5].

### 2.4. Treatment Implications

 Treatment for patients with PDGFRB mutations now centers around minimizing the leukocyte count with imatinib mesylate. This drug is a tyrosine kinase inhibitor, initially developed specifically for the treatment of CML. Imatinib targets the ATP-binding site of BCR-ABL kinase which is the mutated protein product in CML [6, 8]. However, this agent also targets many other kinases, including type 3 transmembrane receptor tyrosine kinases (RTKs) such as Kit and platelet-derived growth factor [5, 6]. The fusion proteins created as a result of PDGFR mutations include active tyrosine kinases that are sensitive to imatinib.

 Some case studies have described durable responses to imatinib treatment at 400 mg daily [5, 6]. Other case reports have discussed the response of PDGFRB fusion MPNs to doses of imatinib which were lower than those typically used to treat CML [2, 8]. Similar findings have also been reported in 14 cases of FIP1L1-PDGFRA patients [3]. A review of 12 cases of PDGFRB-translocation MPNs identified 10 patients with resolution of their cytogenetic abnormalities, as proven by PCR [4]. Similar findings were noted in 14 FIP1L1-PDGFRA patients [3] and 3 ETV6-PDGFRB fusion patients [6]. Four of the FIP1L1-PDGFRA patients who discontinued imatinib suffered relapses, an event also noted in our patient [3]. Doses of imatinib ranged from 100 mg per week to 100 mg daily [3].

## 3. Conclusion

 This report confirms that similar low doses of imatinib have successfully attenuated leukocyte counts in a patient with a PDGFRB mutation. In future cases, lower doses may be indicated to achieve control of this disease. Cytogenetic analysis to verify resolution of this mutation was pending on this patient at the time of publication.
